# Evaluation of the clinical molecule anti-human-PD-L1/IL-15 KD033 in the human-PD-1/PD-L1-expressing murine model demonstrates PD-L1 targeting of IL-15 in vivo

**DOI:** 10.1007/s00262-022-03331-0

**Published:** 2022-12-01

**Authors:** Stella A. Martomo, Jeegar Patel

**Affiliations:** Kadmon Corporation, a Sanofi Company, 450 East 29th Street, New York, NY 10016 USA

**Keywords:** Bi-functional antibodies, Cytokine fusions, Immune-modulation, Tumor-microenvironment

## Abstract

KD033 is a clinical-stage immunocytokine composed of a high-affinity anti-human-PD-L1 antibody and the human IL-15/ IL-15 receptor sushi-domain complex. We have previously shown that KD033-surrogate, the anti-mouse-PD-L1/IL-15 immunocytokine, was efficacious in several syngeneic murine tumor models including those that were refractory to anti-PD-1/PD-L1 checkpoint blockers. KD033-surrogate showed better efficacy than the combination treatment of its component, anti-PD-L1 antibody with the non-targeting IL-15. KD033-surrogate was also efficacious in both low and high PD-L1-expressing tumors. In this study, we have utilized double knock-in mice expressing functional human PD-1/PD-L1 to show that the clinical molecule, KD033, reproduced the anti-tumor efficacy observed with KD033-surrogate in the syngeneic models. KD033 was equally efficacious in reducing the growth of human-PD-L1 positive (hPDL1+) and negative (hPDL1-) MC38 murine tumors. We observed similar peripheral pharmacodynamics changes in KD033-treated mice bearing either hPDL1+ or hPDL1- MC38 tumors. However, different transcriptomic profiles were observed between KD033-treated hPDL1+ and hPDL1- MC38 tumors with marked changes involving mostly downregulated genes in hPDL1- tumors in addition to the immune-related genes changes observed in both hPDL1+ and hPDL1- MC38 tumors. Cytotoxic and myeloid cell signatures were upregulated in both tumors with relatively greater increases observed in hPDL1- MC38 tumors. These effects of KD033 treatment in PD-L1 positive and negative tumors demonstrate the role of PD-L1 in targeting of IL-15 cytokine in vivo.

**Supplementary Information:**

The online version contains supplementary material available at 10.1007/s00262-022-03331-0.

## Introduction

Immunocytokine generated from the fusion of a functional checkpoint antibody and an immune modulating cytokine has become a promising therapeutic modality in immuno-oncology [1]. It has been shown that combination therapies of IL-15 or IL-2 administration with PD-1/PD-L1 blockade was efficacious in murine cancer and infection models [2, 3]. Nevertheless, safety concerns and the short half-life of IL-2 and IL-15 prompted exploration on modifications of these cytokines for more effective therapeutic use. Reducing the potency of IL-15 increases its safety and half-life in serum; however, reduced-potency IL-15 was not efficacious when administered as a free cytokine [4]. IL-2 modifications focus mainly on modulations of its binding to IL-2 receptor alpha [IL-2Rα]. IL-2 lacking IL-2Rα binding has less effect on regulatory T cells expansion and showed increased efficacy with some improvements on its safety [5, 6]. Immunocytokine represents another modality of cytokine modifications. Immunocytokines consisting of IL-15 or IL-2 fused with an anti-PD-1 or anti-PD-L1 checkpoint antibody have the advantage of possessing the immune-stimulatory and checkpoint blockade capabilities in one molecule. Administration of these IL-2 or IL-15 immunocytokines increased the safety and efficacy of the unconjugated cytokines and checkpoint antibody monotherapies or combinations in preclinical tumor models [4, 7, 8].

KD033 is an immunocytokine currently being tested in clinical trials consisting of an anti-PD-L1 antibody conjugated to the wild-type IL-15. KD033 targets IL-15 to PD-L1 positive cells, and in the tumor-microenvironment (TME) this would include both immune and tumor cells. We showed previously that the mouse cross-reactive KD033-surrogate had increased safety compared to the non-targeting IL-15 and significant anti-tumor activity in mouse tumor models that are non- or minimally responsive to IL-15 or anti-PD-L1 monotherapy [8]. A single-dose of KD033-surrogate showed increased efficacy when compared to repeat doses of anti-PD-L1 alone, or the highest tolerated dose of the non-targeting IL-15 in the murine CT26 colon carcinoma model [8]. With the availability of the double knock-in of human-PD-1 and PD-L1 C57/Bl6 mice and the corresponding human-PD-L1-expressing murine tumor cell lines, we tested the efficacy of the clinical molecule, KD033, in this transgenic murine model. We observed previously that KD033-surrogate was efficacious in tumors that expresses varied levels of PD-L1 [8]. Utilizing this human-PD-1/PD-L1 knock-in mice and cell lines, we were able to further study and compare KD033 responses in the same murine tumor cell line with or without human PD-L1 expression. We used the human-PD-L1-transfected murine colorectal MC38 tumor as the PD-L1-positive and the untransfected MC38 as the hPD-L1 negative tumors as KD033 does not cross-react with murine-PD-L1. KD033 was efficacious in both hPD-L1 positive and negative MC38; however, there were significant transcriptomic differences between KD033-treated hPD-L1 positive and negative MC38 tumors.

## Materials and methods

### IL-15 fusion molecules

The three immunocytokines referred in this study: KD033, a fusion of a high affinity anti-human-PD-L1, KD033-surrogate, a fusion of a high affinity anti-mouse-PD-L1, and ntKD033, a fusion of a non-targeting antibody control, all with human IL-15/IL15Rα complex, have been described previously [8].

### In vivo syngeneic murine tumor studies

Murine in vivo studies were conducted for Kadmon by WuxiAppTec, China. All procedures related to animal handling, care and the treatment in the studies were performed according to the guidelines approved by the Institutional Animal Care and Use Committee (IACUC) of WuXi AppTec in accordance with all standards of the Association for Assessment and Accreditation of Laboratory Animal Care (AAALAC) and of Sanofi policies (tumor volumes, transgenic mice). The human-PD-1/PD-L1 knock-in C57/Bl6 mice were obtained from Biocytogen, China. The syngeneic murine colorectal cell line, MC38, (NTCC-MC38, ATCC) was maintained in DMEM medium supplemented with 10% heat inactivated fetal bovine serum (FBS), 100 U/mL penicillin, 100 μg/mL streptomycin and L-glutamine (2 mM). The human-PD-L1-transfected MC38 (hPDL1+ MC38) was maintained in RPMI 1640 medium with 10% FBS and 1% hygromycin B. Both cell lines were grown at 37 °C, 5% CO_2_ and were routinely evaluated for mycoplasma with MycoAlert^®^ Mycoplasma Detection Kit (LT07-118, Lonza). MC38 or hPDL1+ MC38 cells growing in an exponential growth phase were subcutaneously inoculated into the right flank of hPD-1/PD-L1 C57/Bl6 mice. Assessment of efficacy was evaluated in mice bearing either MC38 and hPDL1+ MC38 tumors with a single intravenous KD033 or KD033-surrogate (*n* = 6 per arm) treatment at 3 mg/kg of when tumors reach 100 mm^3^. This dose was shown to be efficacious for KD033-surrogate [8]. Mode of action study followed the efficacy study with intravenous administration of KD033 at 3 mg/kg (*n* = 6 per arm) when tumors reach 128 mm^3^. At day 6 post treatment, tumors were collected for RNA transcriptional and immunohistochemistry analyses, and peripheral blood for flow cytometry analysis. Serum was collected at day 1 and 6 post treatment for cytokine and chemokine measurements.

### Immunohistochemistry

Immunohistochemistry (IHC) was done by WuxiAppTec, China. OCT blocks of tumor samples were sectioned (4 µm thickness/section), and sections on slides then were warmed at room temperature for 30 min, fixed in ice cold 70% ethanol for 15 min and air dried for 15 min before being processed using Leica EG Bond RX. Antibodies used are listed in Supplementary Table S1. All stained slides were scanned with Leica Aperio VERSA 8, and images were analyzed using the HALO™ image analysis platform. Areas of necrosis were excluded. Total cell numbers (or area) and IHC positive cells (or area) were scored. The IHC score represents the positive to total cell count/area ratio in the examined section.

### Flow cytometry

Flow cytometry analysis was done by WuxiAppTec, China. Freshly collected peripheral blood was diluted 20-fold with 1 × Red Blood Cell Lysis solution (cat# 555899, BD), incubated for 2 min and washed. Cells were stained in 100 µL staining buffer (cat# 00-4222, eBioscience) with addition of purified rat anti-mouse CD16/CD32 (1 µL per well, cat# 553142, BD) in 96-well V-bottom plate with fluorochrome-conjugated antibodies listed in Supplementary Table S1. Stained and washed cells were fixed (fixation buffer, cat# 554655, BD) before then analyzed within 24 h. Data were acquired using BD FACS LSR Fortessa X20 Flow Cytometer and analyzed using FlowJo^®^ software.

### Serum cytokine/chemokine analysis

For in vivo murine cytokine/chemokine analysis, sera were collected on days 1 and 6 post treatment as part of the in-life portion of the study conducted in WuxiAppTec, China, and sent frozen to Kadmon laboratories, NY. Cytokine/chemokine levels were analyzed with the MILLIPLEX^®^ Mouse CD8+ T Cell Magnetic Bead Panel Premixed 15 Plex (MCD8MAG48K-PX15, Millipore Sigma). For cytokine/chemokine analysis of the monocyte-derived human macrophages, supernatant was collected at 24 h post addition of compounds by OcellO B.V, CrownBioscience, the Netherlands, and sent frozen to Kadmon laboratories, NY. Cytokine/chemokine levels were analyzed with the MILLIPLEX^®^ Human Cytokine/Chemokine/Growth Factor Panel A 48 Plex without RANTES (HCYTA-60 K-PX48, Millipore Sigma). Frozen sera were thawed just before use and utilized in the assays without dilutions (neat). All steps were done according to each kit’s instructions. Data were collected using the Luminex xMAP MAGPIX instrument (MAGPX17177721, Millipore, Sigma). Before data acquisition, the instrument was calibrated using MAGPIX calibration kit (MPX-CAL-K25) and verified using MAGPIX verification kit (MPX-PVER-K25) according to the kit's instructions. Data acquisition and analysis were done with the xPONENT 4.2 for MAGPIX software.

### Gene transcription analysis

Tumors were isolated on day 6 after treatment and FFPE preserved at WuxiAppTec, China. Tumor RNA extractions and nanostring RNA hybridizations were conducted by Canopy Biosciences (St. Louis, MO). The NanoString mouse PanCancer IO 360 immune-oncology panel was used for transcriptomic analysis. Data was uploaded to the ROSALIND platform (www.rosalind.bio) and analyzed by Canopy Biosciences. Heat maps and volcano plots for transcriptomes were obtained using the differential gene expression analysis the nSolver Advanced Analysis with the “Optimal” method in the ROSALIND platform. All 6 samples per group were included in all analyses.

### Macrophages cultures and analysis

In vitro study of human macrophages with KD033 was conducted by Ocello, B.V, CrownBioscience, the Netherlands. Isolated human CD14+ myeloid PBMC were plated in myeloid cell-Gel (OcellO B.V.) in 3D in 384 well plates (Greiner µClear, Greiner Bio-One B.V.) and incubated at 37 °C in 5% CO_2_. Cells were treated with either GM-CSF (M1) and M-CSF (M2) for 5 days after which LPS was added for another 24 h. M1 and M2 macrophages were then treated with KD033, ntKD033 or anti-PD-L1 (Kadmon) and controls (OcellO) for 24 h. Supernatants were frozen and sent to Kadmon laboratories for cytokine/chemokine analysis.

### Statistics

For comparison between two groups, an independent sample t-test was used. For comparison between three or more groups, one-way ANOVA with Tukey’s multiple comparisons test was used. Comparison of tumor growth between treated and control groups were performed using RM ANOVA. Tests were done on GraphPad Prism software (version 8.4.3.). Significance is indicated as follows: ns (not significant), *p* < 0.05 (*), *p* < 0.01 (**), *p* < 0.001 (***) and *p* < 0.0001 (****). Error bars are SEM.

## Results

### KD033 treatment induced significant tumor growth inhibition of both hPDL1+ and hPDL1- MC38 tumors

The clinical molecule KD033 binds with high affinity to human PD-L1 and blocks PD-1/PD-L1 as well as PD-L1/CD80 interactions, and the human IL-15/IL-15Rα complex was previously shown to be functional in vivo in mice [8]. It is a modified Fc human IgG1 and showed greatly reduced ADCC and CDC [8]. To evaluate the efficacy of KD033 in both human PD-L1 positive and negative tumors, a single intravenous (IV) injection of 3 mg/kg were administered to human-PD-1/PD-L1 knock-in C57/Bl6 mice subcutaneously transplanted with either human-PD-L1 positive- or negative- MC38 (hPDL1+ or hPDL1- MC38) colorectal cancer cells.

**Fig. 1:**
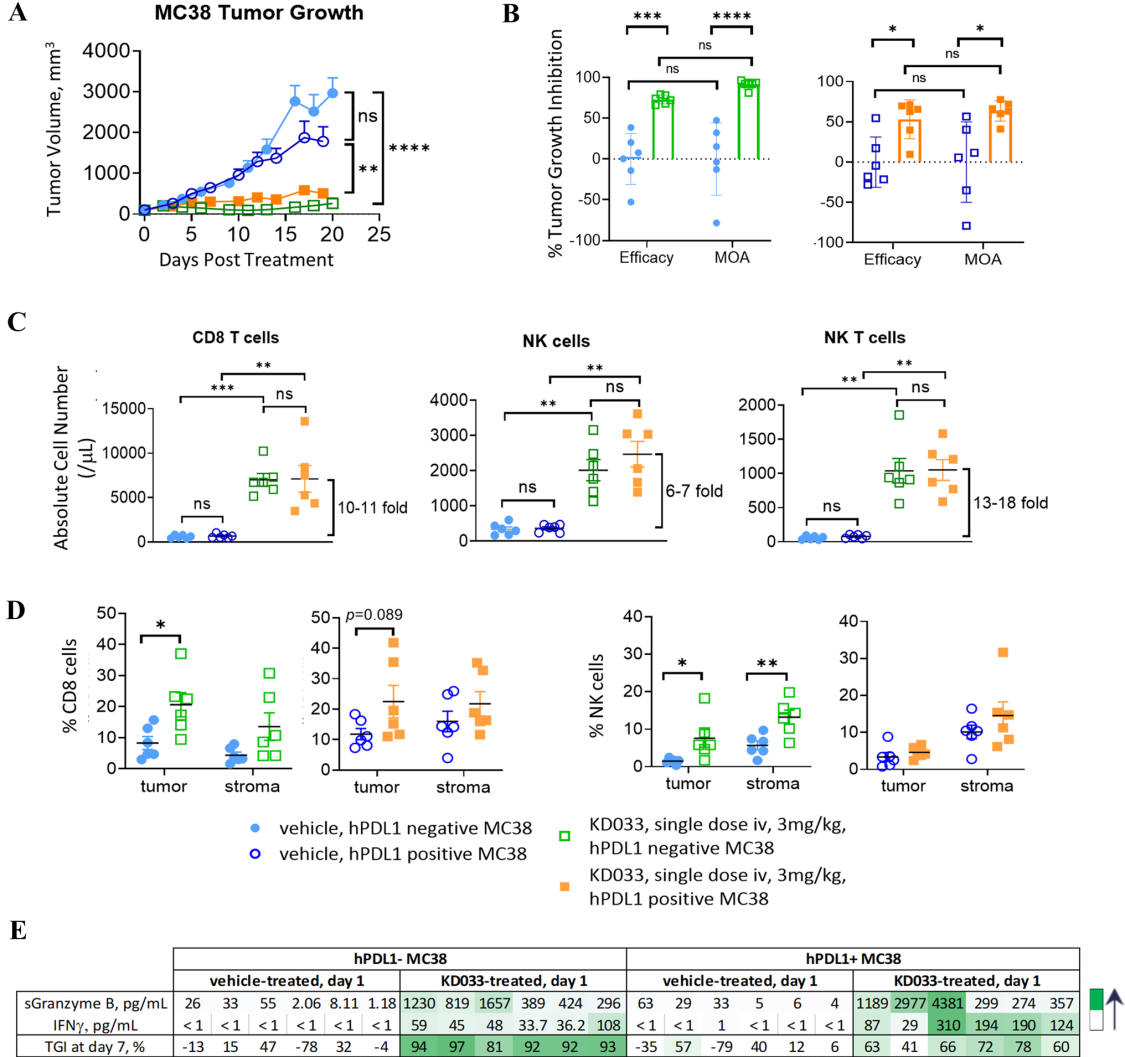
Tumor growth inhibition was observed in human-PD-1/PD-L1 C57Bl/6 mice bearing either hPDL1+ or hPDL1- MC38 tumors after KD033 treatment. **A** Significant tumor-growth inhibition was observed after a single dose of KD033 in both human-PDL1 negative and human-PDL1 positive MC38 (hPDL1- and hPDL1+ MC38) -bearing mice. Significance was calculated with repeat measure ANOVA. **B** Similar tumor growth inhibition for either hPDL1+ or hPDL1- MC38 tumors was observed for the efficacy study (at day 6 or 7 post dose of 3 weeks treatment duration) compared to the following mode of action study (MOA, 6 days post dose). Both studies were done with *n* = 6 per arm. Treatments were administered when tumors reached 100 mm^3^ for the efficacy and 128 mm^3^ for MOA study. **C** Pharmacodynamic changes in peripheral blood 6 days after KD033 treatment with relevant immune cell population measured in absolute count per µL. **D** Immunohistochemistry analysis of tumors collected at day 6 post KD033 administration. **E** Serum analysis from blood collected 24 h post KD033 administration

Treatment with KD033 resulted in significant anti-tumor activity as measured by tumor growth inhibition (TGI) for both hPDL1+ and hPDL1- MC38 (Fig. 1A). This result reproduced the previously observed anti-tumor efficacy of KD033-surrogate (Supplementary Fig. S1A, 8) and validated the previous use of KD033-surrogate to evaluate KD033 efficacy. Repeat dose of anti-PD-L1 antibody was included as comparison (Supplementary Fig. S1A) and showed less effective tumor growth inhibition compared to single injection of KD033 and KD033-surrogate. We followed this KD033 efficacy study with KD033 mode-of-action study (MOA) and showed that the corresponding TGIs of KD033 treatment in these two studies were not significantly different (Fig. 1B, Supplementary Fig. S1A, B and C). The calculated TGIs for the efficacy and MOA studies at day 7 and 6 post treatment for hPDL1- (73% and 91% respectively) was higher than for hPDL1+ MC38 tumors (53% and 63% respectively) (Supplementary Fig. S1C). There was no significant difference between the growth of control hPDL1- and hPDL1+ MC38 tumors indicating similar engraftment of hPDL1- and hPDL1+ MC38 tumors. The average isolated tumor volumes of KD033-treated hPDL1- MC38 was smaller than hPDL1+ MC38 and could indicate a faster kinetics of tumor killing post a single KD033 treatment in hPDL1- compared to hPDL1+ MC38 tumors.

### KD033 treatment induced distinct transcriptomic changes in hPDL1- compared to hPDL1+ MC38 tumors

To assess the pharmacodynamics of KD033 treatment in mice, changes in peripheral blood and tumors were evaluated. Peripheral blood measurement of immune cell populations was in agreement with the result observed previously in KD033-surrogate treated mice and showed no difference between mice bearing hPDL1+ or hPDL1- MC38 tumors (Fig. 1C and 8). Increased absolute count of CD8+ T (~ ten fold), NK (~ six fold) and NK T cells (~ 15 fold) were observed on day 6 post KD033 treatment in both hPDL1+ and hPDL1- MC38 -bearing mice (Fig. 1C). Higher CD8+ T absolute count increases compared to NK cell increases in peripheral blood after KD033 treatment reproduced what was observed previously with KD033-surrogate treatment [8]. Higher percentages of CD8+ T and NK cells in blood of both hPDL1+ and hPDL1- MC38 -bearing mice was also observed after KD033 treatment (Supplementary Fig. S1D), whereas only minimal increases in absolute count of CD4+ T and CD19+ cells were observed (Supplementary Fig. S1E).

In contrast to peripheral immune cell changes, we observed marked differences between hPDL1+ and hPDL1- MC38 tumors treated with KD033. IHC analysis showed increased CD8+ T cell infiltration into the tumors and in the stroma of both hPDL1+ and hPDL1- tumors which reached statistical significance in hPDL1- MC38 tumors (Fig. 1D). Of note, there was a tendency for higher densities of CD8+ T cell in the tumor and stroma in the control hPDL1+ compared to control hPDL1- MC38 tumors. Increased NK cell infiltration into the tumors and in the stroma was only observed in hPDL1- MC38 tumors (Fig. 1D). There were also increases in B220 + and regulatory T cells infiltrations into hPDL1- tumors that was not observed in hPDL1+ tumors (Supplementary Fig. S1F). Increased infiltration of diverse immune cell populations in addition to CD8+ T and NK cells into hPDL1- tumors would be consistent with ongoing inflammation and tumor cell killing at day 6 post KD033 treatment in hPDL1- MC38 tumors.

The changes in serum cytokines and chemokines in the peripheral blood after KD033 treatment was also evaluated. As shown in Fig. 1E, there were increases in IFNγ and soluble granzyme B in the sera of KD033-treated mice 24 h post dose with some hPDL1+ MC38 bearing mice showing marginally higher IFNγ and granzyme B compared to the average increases in hPDL1- MC38 bearing mice. IFNγ increases in the serum was expected as the on-target mechanism of IL-15 [9], and soluble granzyme B indicated cytotoxic cells activation [10]. However, the peripheral serum IFNγ and granzyme B measurements did not closely reflect KD033 activity in the tumors as the magnitude of increases did not correlate to the observed anti-tumor activity levels (Fig. 1E).

**Fig. 2:**
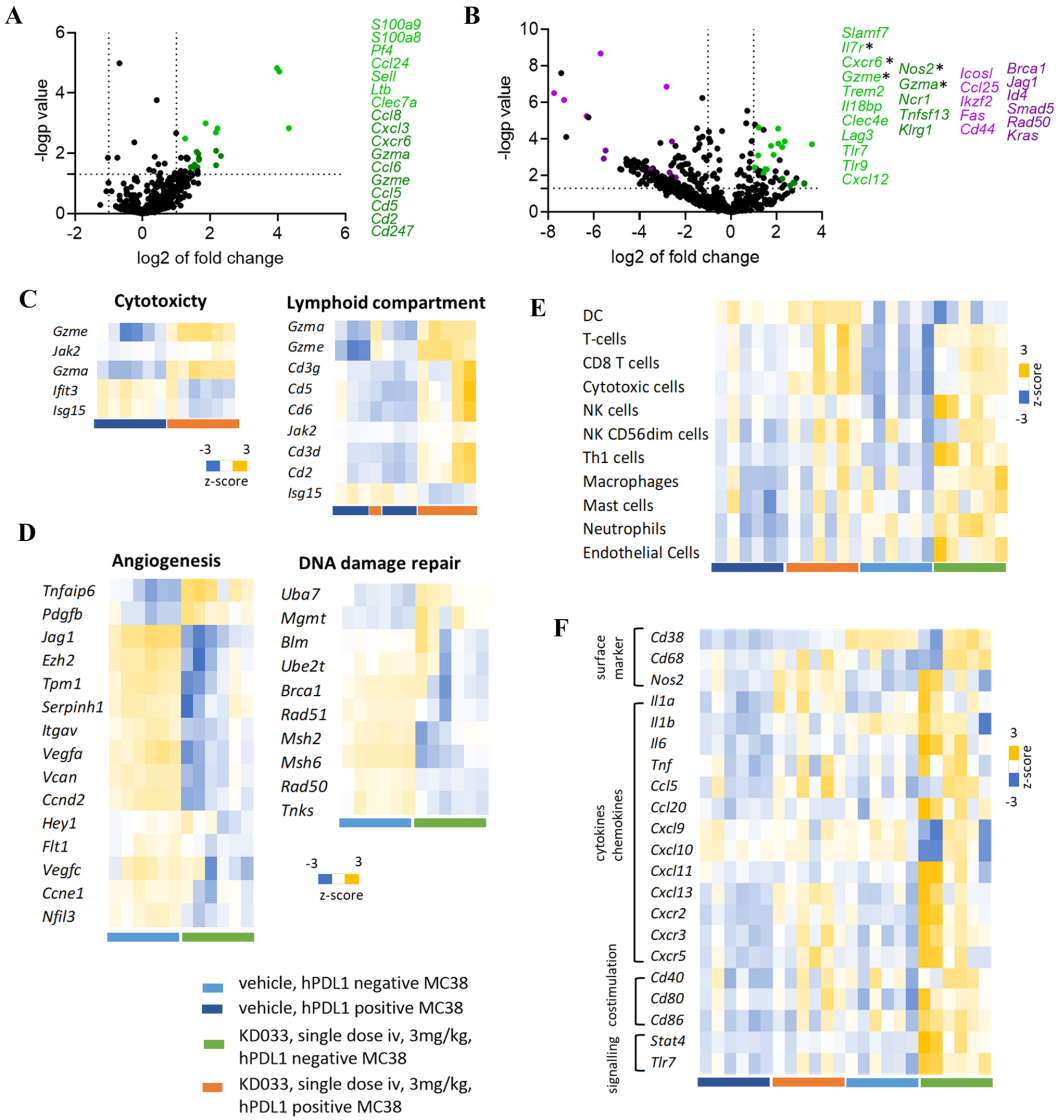
Different hPDL1+ and hPDL1- MC38 tumors transcriptome profiles after treatment with KD033. Tumors transcriptome were analyzed with Nanostring mouse Pan IO360. **A** Volcano plot of hPDL1+ MC38 tumors treated with KD033 versus vehicle showing upregulated genes after KD033 treatment. **B** Volcano plot of hPDL1- MC38 tumors treated with KD033 versus vehicle showing both upregulated and downregulated genes after KD033 treatment with downregulated genes dominating the transcriptome. * denotes genes that were also increased when hPDL1- MC38 transcriptome was compared to hPDL1+ MC38. **C**, **D** Top first and second pathways identified after treatment with KD033 for hPDL1+ MC38 tumors (C) and hPDL1- MC38 tumors (D). **E** Comparison of different immune cell populations in tumors identified by the nanostring cell-type profiler. **F** Comparison of select macrophage related genes

Transcriptomic analysis using the Nanostring gene expression platform showed marked differences between KD033-treated hPDL1+ and hPDL1- MC38 tumors (Fig. 2). Volcano plot of transcriptome from KD033- versus vehicle- treated hPDL1+ MC38 tumors showed a small number of significantly changed genes, most were upregulated after KD033 treatment (Fig. 2A and Supplementary Fig. S2A). Some of these significantly upregulated genes are listed in Fig. 2A with changes mostly reflected in genes involved in the immune response, such as cytotoxicity (*Gzma, Gzme*), T cells (*Cd5, Cd2, Cd247*), chemokines (*Ccl24, Ccl8, Ccl6, Cxcl3, Cxcr6*) and inflammation (*S100a9, S100a8*). These changes are in agreement with the on-target mode of action of KD033 as the result of KD033 binding to tumor cells to directly activate cytotoxic CD8+ T cells in the TME.

Volcano plot of KD033-treated versus control hPDL1- MC38 showed a large number of significant gene transcription changes with both upregulated and downregulated genes (Fig. 2B and Supplementary Fig. S2B). It is interesting to note that while most of the upregulated genes were immune related genes (*Il7r, Cxcr6, Gzme, Tlr7, Tlr9, Gzma, Klrg1*), the downregulated genes were more diverse consisting of some immune related genes (*Icosl, Ccl25, Fas*) and many more genes in other pathways such as DNA repair (*Rad50, Kras, Brca1*) (Fig. 2B). Volcano plot of the vehicle-treated hPDL1- versus hPDL1+ MC38 tumors did not show these changes (Supplementary Fig. S2C). Furthermore, there were only several genes that were transcriptionally altered by more than two-fold when vehicle-treated hPDL1- to hPDL1+ MC38 tumors were compared, indicating similar transcriptional profiles between the tumors without KD033 treatment.

Pathway analysis of transcriptomic changes in KD033-treated versus control hPDL1+ tumors resulted in two pathways: cytotoxity and lymphoid compartment, as the first and second pathways affected respectively (Fig. 2C). This is in agreement with what was observed previously in KD033-surrogate treated CT26 tumors, and activation of cytotoxic cells in TME when KD033 binds to tumor cells. The same analysis in KD033-treated versus control hPDL1- tumors resulted in completely different pathway changes with angiogenesis and DNA repair pathways as the first and second pathways affected respectively (Fig. 2D). These two pathways were downregulated significantly in KD033-treated hPDL1- tumors indicating possible KD033-directed and immune-cell mediated, tumor cell-intrinsic changes.

Nanostring immune cell profiler showed that control hPDL1+ MC38 tumors had more T cells and dendritic cells compared to hPDL1- MC38 which had higher levels of myeloid cells in the tumor (Fig. 2E). Higher CD8+ T cells in control hPDL1+ MC38 tumors confirmed what was observed also by IHC (Fig. 1D). After KD033 treatment, both hPDL1+ and hPDL1- MC38 tumors showed increased T, cytotoxic and CD8+ T cells infiltrations. Higher levels of NK and Th1 cells as well as myeloid cells infiltrations were observed in hPDL1- compared to hPDL1+ MC38 tumors (Fig. 2E). Macrophage was one of the myeloid cell populations increased after KD033 treatment in hPDL1- tumors, and expression of macrophage-related genes were evaluated further (Fig. 2F). Select macrophage-related genes involved in signaling, co-stimulation, chemokines/cytokines pathways as well as some surface marker genes were upregulated in KD033-treated hPDL1- tumors while these same genes were not significantly changed in KD033-treated hPDL1+ tumors.

### Functional and morphological changes in macrophages with KD033 exposure

**Fig. 3:**
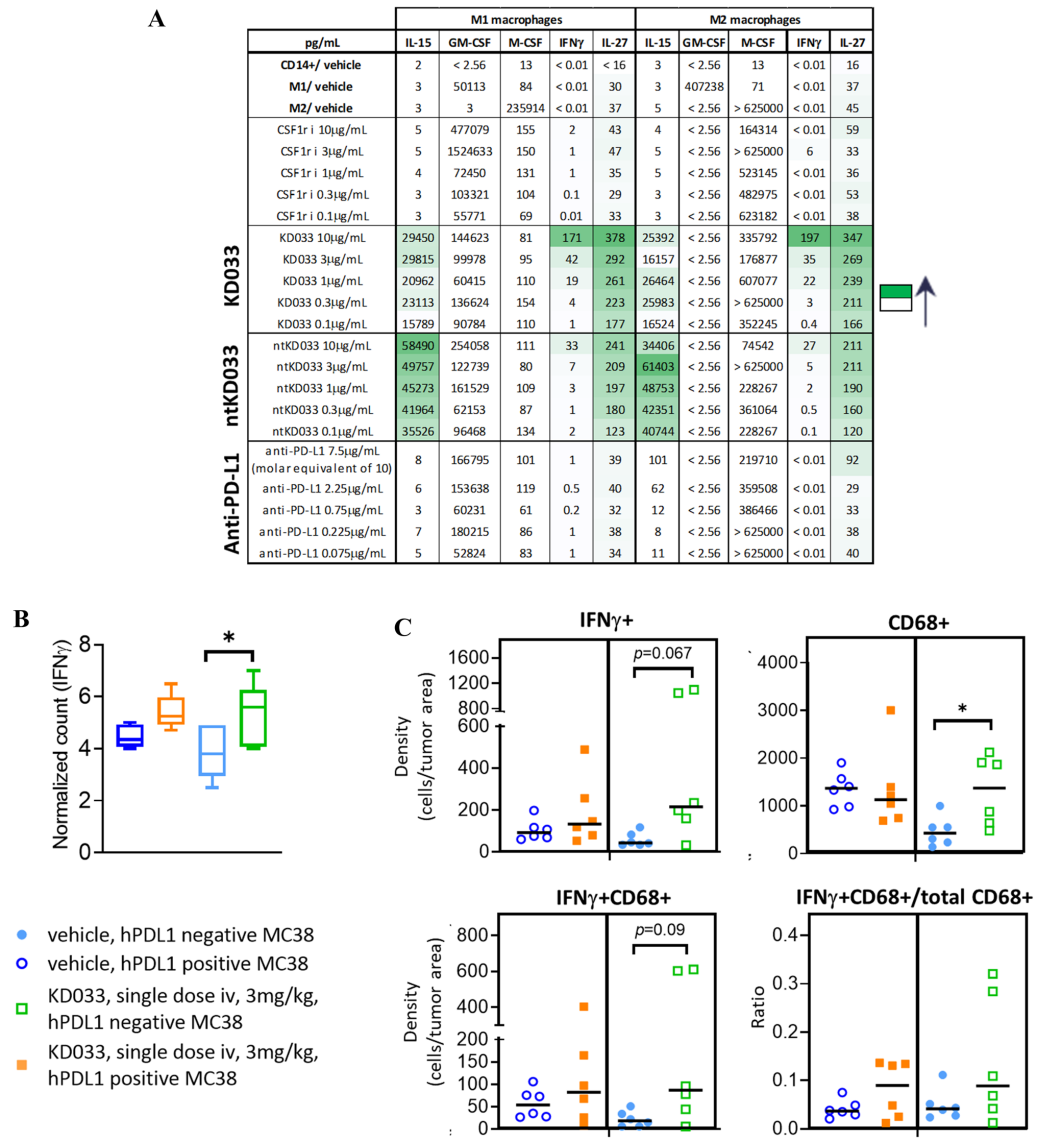
KD033 induces phenotypic and functional changes in macrophage in vitro **A** IFNγ was secreted from human monocyte-derived M1 and M2 macrophages cultured in 3D spheres with KD033 for 24 h. **B** Significant increase in IFNγ gene transcription in hPDL1- MC38 tumors compared to vehicle control at day 6 post treatment. **C** Immunohistochemistry of IFNγ and CD68 in KD033 treated hPDL1+ and hPDL1- MC38 tumors at day 6 post treatment

KD033 binds in a dose-dependent manner to human monocyte-derived M1 and M2 macrophages (Supplementary Fig. S3A). This binding is most likely through PD-L1, as the non-targeting control antibody IL-15 fusion protein (ntKD033) does not show dose-dependent binding to macrophages. Both KD033 and ntKD033 have Fc modification; therefore, were not expected to bind non-specifically to macrophages. It was shown previously that anti-PD-L1 antibody can directly affect macrophages [11]. In vitro evaluation of KD033 on isolated macrophages can provide evidence of a similar direct effect. After KD033 was added to human M1 and M2 macrophages cultured in 3D spheres for 24 h, some functional and morphological changes were observed (Fig. 3A and Supplemental Fig. S3B). As shown in Fig. 3A, increased in IFNγ and IL-27 was observed in supernatants of both M1 and M2 macrophages cultured with KD033 but not when they were cultured with anti-PD-L1 antibody. Some increased IFNγ and IL-27 was also observed in supernatants of macrophages cultured with ntKD033; however, the magnitude of increase was much less, and KD033 was more efficient than ntKD033 at inducing IFNγ and IL-27 secretions. Very high concentrations of IL-15 in the supernatants were observed in macrophages cultured with either KD033 or ntKD033 which reflected the cross-reactivity of the IL-15 antibodies used in the detection kit to both KD033 and ntKD033 fusion proteins added to the cultures. Since KD033 and ntKD033 were both added at the same concentrations, the lower serum IL-15 detection in the supernatants of KD033-treated compared to the corresponding ntKD033-treated macrophage cultures (Fig. 3A) can be explained by the binding of KD033 to the surface of 3D-cultured macrophages. We also observed phenotypic changes in M1 macrophages which became more irregular in shape after 24 h of culture with KD033 (Supplementary Fig. S3B), ntKD033 and anti-PD-L1, but not with vehicle (data not shown). However, repolarization of M2 into M1 macrophages was not observed after 24 h of treatment with either KD033, ntKD033 or anti-PD-L1 antibody (data not shown).

To evaluate if KD033 can similarly affect macrophages in tumors in vivo, KD033-treated tumors sections were evaluated for IFNγ and CD68 through IHC. Increased in IFNγ gene transcription was significant for hPDL1- when compared to control MC38 tumors with a tendency of control hPDL1- having less IFNγ compared to control hPDL1+ MC38 tumors (Fig. 3B). Similarly, when analyzed by IHC, increased IFNγ density was observed mainly in KD033-treated hPDL1- tumors. Furthermore, control hPDL1- had slightly less IFNγ compared to control hPDL1+ MC38 tumors (Fig. 3C). Significant increase in CD68 infiltration was observed in hPDL1- tumors; however, CD68 positivity was already relatively high in both control and KD033-treated hPDL1+ MC38 tumors. Increased IFNγ + CD68 + double positivity was observed in both hPDL1+ and hPDL1- MC38 tumors with a higher increase observed in hPDL1- tumors. Increased proportion of INFγ + CD68 + among total CD68 + cells was also observed in hPDL1- tumors although this increase did not reach significance. To summarize, activation of macrophages seemed to play a major role in anti-tumor activity of KD033 in hPDL1- tumors as shown in increased expression of genes involved in macrophage signaling, co-stimulation and pro-inflammatory cytokines/chemokines secretion in addition to some evidence of direct effect of KD033 on macrophages in vitro*.*

## Discussion

We have evaluated the efficacy and mode of action of the clinical molecule KD033 in the human PD-1/PD-L1- expressing mice bearing either hPDL1+ or hPDL1- MC38 murine colon carcinoma tumors. KD033 anti-tumor activity reproduced the previously reported KD033-surrogate efficacy in syngeneic murine models: a single injection of KD033 was more efficacious than the repeat treatment of anti-PD-L1, and KD033 was efficacious in both hPDL1+ and hPDL1- MC38 tumors. We have designed KD033 to target IL-15 to PD-L1+ tumors and to bring cytotoxic cells to the tumor-microenvironment; therefore, the observation that KD033 and KD033-surrogate had significant anti-tumor efficacies and comparable tumor-growth inhibitions for both PD-L1+ and PD-L1- tumors was unexpected. Furthermore, as KD033 is a clinical molecule, its significant anti-tumor activity in PD-L1- tumors would greatly impact the tumor target selections in the clinical study. In this report, we have focused our analysis on understanding KD033 mode of action in PD-L1+ compared to PD-L1- tumors using the double knock-in of human-PD-1/PD-L1 murine model.

KD033 stimulated intra-tumoral cytotoxicity responses in both human PD-L1- and PD-L1+ MC38 tumors; however, KD033 treatment resulted in different tumor transcriptomic changes in hPDL1+ compared to hPDL1- MC38 tumors. Though increased cytotoxic and lymphoid cell genes were observed in both KD033-treated hPDL1+ and hPDL1- MC38 tumors, KD033-treated hPDL1- tumors showed decreases in angiogenesis and DNA repair pathways. These distinct KD033-induced transcriptomic changes between PD-L1 positive and negative tumors can represent two divergent dominant mode of actions of KD033 in TME.

We showed dose-dependent binding of KD033 to human monocyte-derived macrophages and propose that KD033 binds to PD-L1+ myeloid cells in the TME of PD-L1- tumors. In vitro, KD033 induced human monocyte-derived macrophages to secrete pro-inflammatory cytokines more efficiently than the non-targeted, free IL-15, whereas anti-PD-L1 did not induce pro-inflammatory cytokine secretion. In PD-L1- tumors, KD033 treatment seemed to induce increased pro-inflammatory cytokine/chemokine secretions and myeloid cells signatures with increased cytotoxic cell trafficking including increased NK cells into the TME. Pro-inflammatory TME can also affect tumor cells intrinsic pathways such as tumor cell DNA repair pathway, which in turn would result in fast kinetics of tumor-growth inhibition. In theory, this hypothesis can be tested using in vivo macrophage depletion coupled with either M1 and/or M2 reconstitution [12]; however, in practice the result of such experiment could be difficult to interpret. In vivo macrophage depletion through anti-CSFR1 antibody was reported to result in varying degree of depletion [13] and clodronate-liposome treatment would deplete dendritic cells as well as macrophages [12].

In PD-L1+ tumors, we argue that KD033 binds mostly to tumors compared to PD-L1+ immune cells present in the TME. The binding of KD033 should be determined by the relative PD-L1 expression on tumors versus immune cells and the relative abundance of PD-L1+ tumors compared to immune cells. For PD-L1+ MC38 TME in this study, it is likely that after a single injection KD033 was bound to PD-L1+ tumors instead of PD-L1+ immune cells which would diminish the role of KD033 in activating PD-L1+ macrophages in the TME. The binding of KD033 to PD-L1+ tumor cells seemed to focus the IL-15 responses as KD033 could act as the costimulatory signal and checkpoint blocker for CD8+ T cells when KD033is bound to PD-L1+ tumor cells. The increased in CD8 T cell infiltration into tumors (Fig. 1C), in cytotoxic and T cell gene signatures, as well as the prevalence of dendritic cell gene signature in h PD-L1+ MC38 tumors (Fig. 2E) suggested that cytotoxic CD8 T cells were the main immune cell population responsible for the anti-tumor activity of KD033 in PD-L1+ tumors. Activation of CD8+ T cells in TME in PD-L1+ tumors constituted a slower tumor killing kinetics relative to NK cell tumor killing in the context of the pro-inflammatory environment of KD033-treated PD-L1- tumors. In agreement to this, at day 6 post KD033 treatment, isolated hPDL1- tumors were smaller compared to hPDL1+ MC38 tumors. This data provided evidence for PD-L1 targeting of IL-15 in vivo as free IL-15 would be expected to have similar mode of action in either PD-L1+ or PD-L1- tumor microenvironment.

In conclusion, in this report we showed that KD033, the anti-human-PD-L1/IL-15 molecule currently in clinical study, had similar anti-tumor efficacies as its mouse surrogate, the anti-mouse-PD-L1/IL-15. We observed that single dose KD033 was more efficacious than repeat dose anti-PD-L1 monotherapy which is similar to what was shown previously of single dose KD033-surogate [8]. Furthermore, KD033-surrogate was effective in tumors with varied PD-L1 expression levels. To evaluate possible tumor markers for KD033 clinical study, we compared KD033 efficacies and mode of actions in human-PD-L1 positive and negative tumor models. We observed significant KD033 anti-tumor efficacies in both PD-L1+ and PD-L1- tumors with differences in transcriptomes of h PD-L1+ versus hPD-L1- KD033-treated tumors. PD-L1 expression on either tumor or myeloid cells in the TME can be tested as a predictive biomarker for KD033 treatment. We propose that the divergent anti-tumor activities of KD033 in PD-L1- and PD-L1+ tumors provide evidence for PD-L1 targeting of IL-15 in vivo*.* Free, non-targeted IL-15 should result in the same mode of action for either PD-L1+ or PD-L1- tumors. Therefore, the PD-L1 targeting of IL-15 differentiates KD033 treatment from ICIs, free IL-15, modified free IL-2, or immune-checkpoint inhibitor treatments, and highlights the merit of KD033’s testing in various cancer indications.

## Supplementary Information

Below is the link to the electronic supplementary material.

Supplementary file1 (PDF 550 KB)

## Data Availability

The data generated and analyzed will be made from the corresponding author on reasonable request.
