# Supplementary material for: Evaluation of the clinical molecule anti-human-PD-L1/IL-15 KD033 in the human-PD-1/PD-L1-expressing murine model demonstrates PD-L1 targeting of IL-15 in vivo
Source: Cancer Immunol Immunother. 2022 Dec 1;72(6):1941–50. doi: 10.1007/s00262-022-03331-0 (PMC10198867; doi:10.1007/s00262-022-03331-0)
Supplement: Supplementary file 1 — Supplementary file1 (PDF 550 KB) [file 262_2022_3331_MOESM1_ESM.pdf]

Supplementary Figure 1. Significant KD033 anti-tumor activity observed in the efficacy study was reflected in the subsequent mode of action study. A. Anti-tumor activity of KD033 was observed in both human-PD-L1 positive and negative MC38- bearing human-PD-1/PD-L1 knock-in C57/Bl6 mice. Similar TGI was observed for KD033 and KD033-surrogate in hPDL1+ MC38. KD033-surrogate cross-react with human PD-L1. Repeat dose anti-PD-L1 antibody is included in the right panel. **B.** Mode of action study was performed subsequently for both models and showed similar tumor regressions corresponding to what were previously observed on days 6 and 7 of the efficacy study. Dashed lines correspond to the same time-point in corresponding studies. **C**. Comparison of tumor volumes and calculated TGIs for the efficacy and MOA studies. Smaller hPDL1- compared to hPDL1+ tumors were isolated. **D**. Immune cell measurements (in percentages of CD45+ cells) in peripheral blood on day 6 post KD033 treatment showing similar increases after treatment. **E.** Absolute count increases in CD4+ T and B cells in peripheral blood after KD033 treatment. **F**. Immunohistochemistry showed some increases in B220+ and FoxP3+ cells after KD033 treatment in human-PD-L1 negative MC38 tumors.

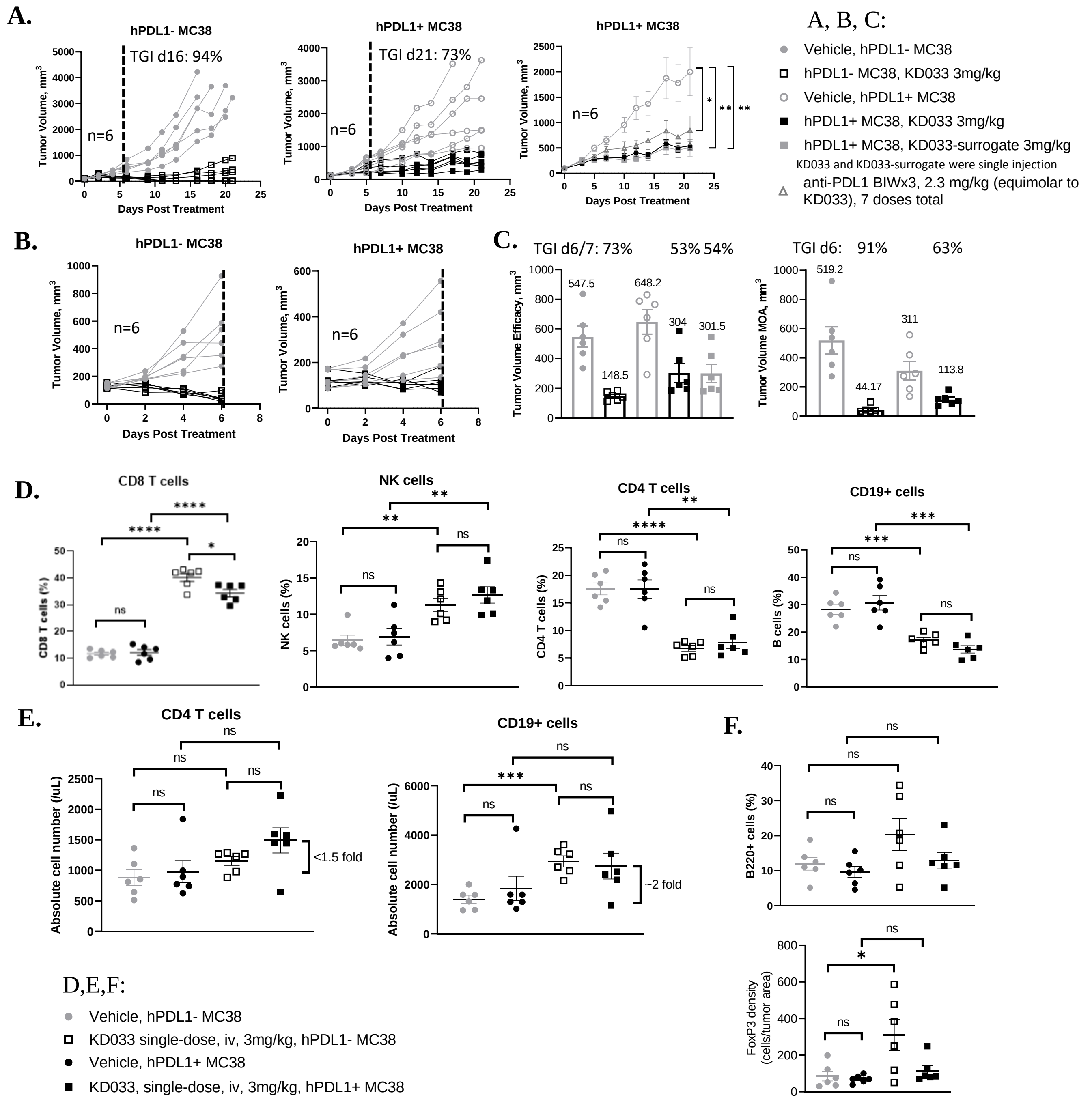

Supplementary Figure 2. Nanostring analysis of FFPE-preserved tumors 6 days after KD033 treatment with the corresponding individual tumor-growth inhibition (TGI) shown. **A**. hPD-L1+ MC38 tumors showed mostly increased gene transcriptions after KD033 treatment with one of the vehicle-treated tumors with a high TGI clustering with the KD033-treated tumors. Only a few genes were transcriptionally downregulated. **B.** More genes were affected by KD033 treatment in hPDL1- MC38 tumors with the majority of the genes transcriptionally downregulated compared to vehicle-treated tumors. A clear distinction was observed between KD033-treated and vehicle-treated tumors. At day 6 after treatment, the volumes of hPDL1- MC38 tumors were small as TGIs for this group were mostly >90%. Increased gene transcriptions were indicated by the green bar and downregulated genes by the purple bar at the bottom. Vehicle-treated tumors were indicated by the black bar and KD033-treated tumors by the red bar at the side. Z-score of genes with significant changes were used to generate the heatmaps. **C**. Volcano plot of vehicle-treated hPDL1- versus hPDL1+ MC38 tumors showed few genes that were transcriptionally changed by more than 2 fold.

A.

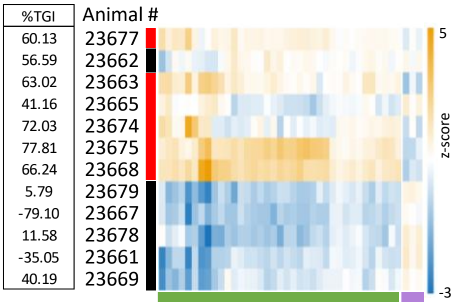

B.

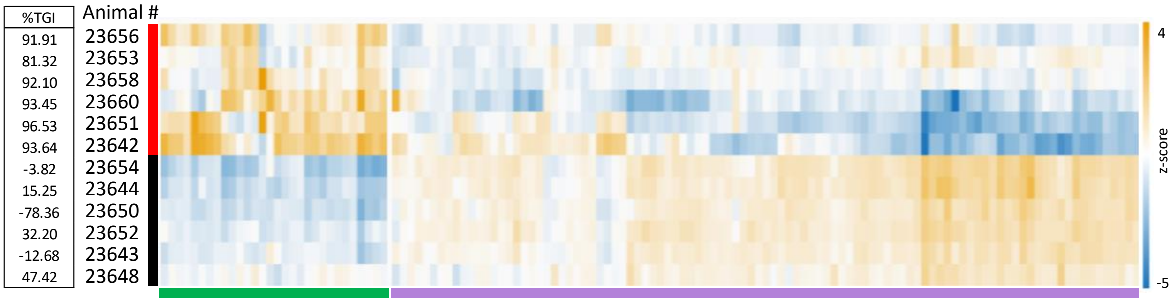

C.

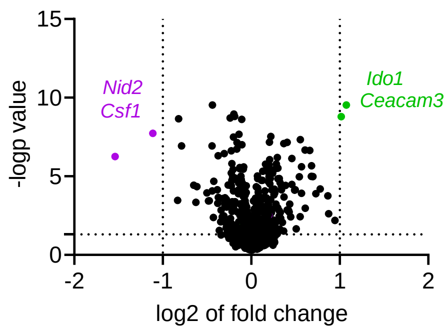

Supplementary Figure 3. A. KD033 and anti-PD-L1 antibody bind to human monocyte-derived macrophages *in vitro*. PBMC was isolated from LeukoPak using Ficoll density gradient centrifugation and monocytes were isolated from PBMC using the EasySep Human Monocyte Enrichment Kit. Isolated monocytes was cultured in RPMI-1640+10% FBS and human GM-CSF (100ng/ml) or M-CSF (100ng/ml) for M1 and M2 respectively for 3 days. Half of the medium was changed with fresh GM-CSF or M-CSF (100ng/ml) at day 3. At day 5, GM-CSF (100ng/ml) + IFN-gamma (50ng/ml) or M-CSF (100ng/ml) + IL-4 (20ng/ml) were added toM1 and M2 cultures respectively to further mature the polarized macrophages. Binding was analyzed with flow cytometry.. **B**. Morphological changes in M1 and M2 macrophages cultured in 3D with KD033 for 24 hours. 3D macrophage cultures were as described in Materials and Methods. Cells were fixed with 4% Formaldehyde (Sigma Aldrich) and simultaneously permeabilized with Triton-X100 (Sigma Aldrich) and stained with Rhodamine-Phalloidin (Sigma Aldrich) and Hoechst 33258 (Sigma Aldrich) in 1x PBS overnight at 4°C, protected from light. After fixation and staining, plates were washed with PBS and sealed with a Greiner SilverSeal (Greiner Bio-One B.V.). Imaging was done using Molecular Devices ImageXpress Micro XLS (Molecular Devices) with a 4x NIKON objective. For each well, multiple images in the z-direction were made for both channels, capturing the whole z- plane. Image analysis was performed using Ominer® software (OcellO B.V.). Individual myeloid cells were segmented using detection of Hoechst-stained nuclei and Rhodamine-Phalloidin-stained cellular f-actin. These segmentation masks were used to analyze the total cell number and phenotypic profile of the myeloid cells per well. The most distinguishing characteristics separating each differentiated group were identified (size and shape) and a cluster analysis was performed with these features which created phenotypic profiles for each cell population. A similarity score was calculated of each treated condition comparing to clusters of untreated CD14+ monocytes and polarized M1 and M2 macrophages.

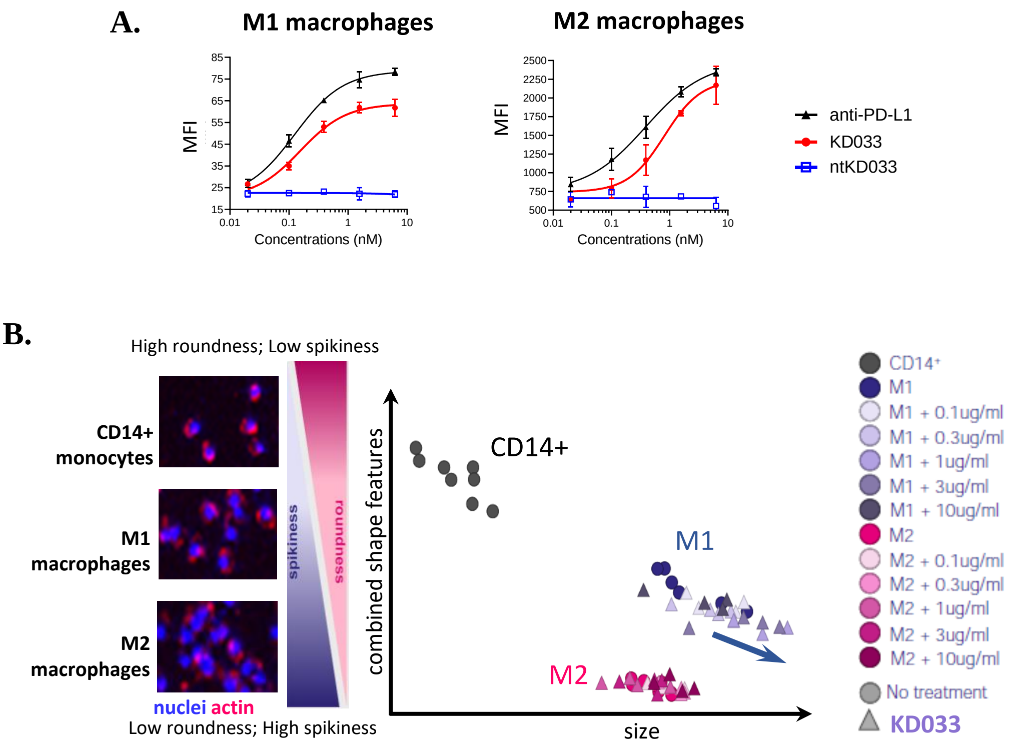

Supplementary Table 1. Antibodies used in flow cytometry and immunohistochemistry

Markers	Fluorochrome	Cat #	Vendor	Note
CD8	BUV395	563786	BD	FC
CD4	BUV496	564667	BD	FC
CD3	APC-Cy7	560590	BD	FC
CD19	BV605	563148	BD	FC
CD45	FITC	553080	BD	FC
CD49b	PE	553858	BD	FC
CD3	APC-Cy7	560590	BD	FC
Live/Dead	BV421	L34964	Invitrogen	FC
CD8	N/A	98941	CST	IHC
B220	N/A	550286	BD	IHC
NK1.1	N/A	39197	CST	IHC
Human Fc	N/A	ab98616	Abcam	IHC
CD68	N/A	ab125212	Abcam	IHC
IFN gamma	N/A	PA5-95560	ThermoFischer	IHC
Goat IgG	N/A	I-5000-5	Vector	IHC
Rabbit IgG	N/A	ab172730	Abcam	IHC

FC: Flow Cytometry

IHC: Immunohistochemistry
